# Bevacizumab, Irinotecan, and Biweekly Trifluridine/Tipiracil for Metastatic Colorectal Cancer: MODURATE, a Phase Ib Study

**DOI:** 10.1093/oncolo/oyad143

**Published:** 2023-06-07

**Authors:** Hiroya Taniguchi, Kentaro Yamazaki, Toshiki Masuishi, Takeshi Kawakami, Yusuke Onozawa, Kazunori Honda, Shigenori Kadowaki, Yukiya Narita, Takahiro Tsushima, Satoshi Hamauchi, Akiko Todaka, Tomoya Yokota, Masashi Ando, Keita Mori, Hiromichi Shirasu, Hirofumi Yasui, Kei Muro

**Affiliations:** Department of Clinical Oncology, Aichi Cancer Center Hospital, Aichi, Japan; Division of Gastrointestinal Oncology, Shizuoka Cancer Center, Shizuoka, Japan; Department of Clinical Oncology, Aichi Cancer Center Hospital, Aichi, Japan; Division of Gastrointestinal Oncology, Shizuoka Cancer Center, Shizuoka, Japan; Division of Gastrointestinal Oncology, Shizuoka Cancer Center, Shizuoka, Japan; Department of Clinical Oncology, Aichi Cancer Center Hospital, Aichi, Japan; Department of Clinical Oncology, Aichi Cancer Center Hospital, Aichi, Japan; Department of Clinical Oncology, Aichi Cancer Center Hospital, Aichi, Japan; Division of Gastrointestinal Oncology, Shizuoka Cancer Center, Shizuoka, Japan; Division of Gastrointestinal Oncology, Shizuoka Cancer Center, Shizuoka, Japan; Division of Gastrointestinal Oncology, Shizuoka Cancer Center, Shizuoka, Japan; Division of Gastrointestinal Oncology, Shizuoka Cancer Center, Shizuoka, Japan; Department of Clinical Oncology, Aichi Cancer Center Hospital, Aichi, Japan; Clinical Research Center, Shizuoka Cancer Center, Shizuoka, Japan; Division of Gastrointestinal Oncology, Shizuoka Cancer Center, Shizuoka, Japan; Division of Gastrointestinal Oncology, Shizuoka Cancer Center, Shizuoka, Japan; Department of Clinical Oncology, Aichi Cancer Center Hospital, Aichi, Japan

**Keywords:** bevacizumab, chemotherapy, colorectal cancer, irinotecan, trifluridine/tipiracil

## Abstract

**Background:**

In this phase Ib study MODURATE, we optimized the dosing schedule and tested the efficacy and safety of trifluridine/tipiracil, irinotecan, and bevacizumab in patients with metastatic colorectal cancer with fluoropyrimidine and oxaliplatin treatment failure.

**Methods:**

We included a dose escalation (3 + 3 design) and an expansion cohort. Patients were administered trifluridine/tipiracil (25-35 mg/m^2^ twice daily, days 1-5), irinotecan (150–180 mg/m^2^, day 1), and bevacizumab (5 mg/kg, day 1) every 2 weeks. The recommended phase II dose (RP2D) in the dose escalation cohort was administered to at least 15 patients in both cohorts combined.

**Results:**

Twenty-eight patients were enrolled. Five dose-limiting toxicities were observed. RP2D was defined as trifluridine/tipiracil 35 mg/m^2^, irinotecan 150 mg/m^2^, and bevacizumab 5 mg/kg. Of 16 patients who received RP2D, 86% (14/16) experienced grade ≥3 neutropenia without febrile neutropenia. Dose reduction, delay, and discontinuation occurred in 94%, 94%, and 6% of patients, respectively. Three patients (19%) showed partial response and 5 had stable disease for >4 months, with a median progression-free and overall survival of 7.1 and 21.7 months, respectively.

**Conclusion:**

Biweekly trifluridine/tipiracil, irinotecan, and bevacizumab administration may have moderate antitumor activity with high risk of severe myelotoxicity in previously treated patients with metastatic colorectal cancer [UMIN Clinical Trials Registry (UMIN000019828) and Japan Registry of Clinical Trials (jRCTs041180028)].

## Teaser

In this phase Ib study, MODURATE, efficacy and safety of trifluridine/tipiracil, irinotecan, and bevacizumab was tested in patients with metastatic colorectal cancer with fluoropyrimidine and oxaliplatin treatment failure

**Lessons Learned**

- The recommended dose was determined as trifluridine/tipiracil of 35 mg/m^2^ twice daily, days 1-5, plus irinotecan of 150 mg/m^2^ combined with BEV of 5.0 mg/kg on day 1 for 2 weeks.
- Biweekly trifluridine/tipiracil, irinotecan, and bevacizumab administration may have moderate antitumor activity with high risk of severe myelotoxicity in previous treated metastatic colorectal cancer patients.

## Discussion

Refractory metastatic metastatic colorectal cancer (mCRC) treatment with trifluridine/tipiracil (FTD/TPI) combined with bevacizumab (BEV) or irinotecan (IRI) showed enhanced therapeutic effects in preclinical models.^1,2^ A phase Ib study combining standard FTD/TPI regimens with irinotecan (IRI) reported antitumor activity but high degree of febrile neutropenia.^3^ Hence, doses of this combination require optimization. It was recently reported that a regimen of FTD/TPI twice daily biweekly for days 1-5 yielded efficacy similar to that of standard scheduling (twice daily for days 1-5 and again on days 8–12, repeated monthly), but with reduced toxicity.^4^ Therefore, we designed a phase Ib study to establish an optimal dosing schedule and explore the safety and efficacy of biweekly FTD/TPI administration together with IRI and BEV in mCRC patients unresponsive to fluoropyrimidine and oxaliplatin.

In 18 patients of the dose escalation part, 5 DLTs occurred. We determined that the recommended phase II dose (RP2D) could be defined as FTD/TPI 35 mg/m^2^ plus IRI 150 mg/m^2^ combined with BEV (5.0 mg/kg) for 2 weeks, and additional 10 patients were enrolled at this RP2D. Drug-related AEs occurred in all patients; mostly grades 3-4 neutropenia (86%) that could be managed by delay in the treatment schedule, dose reduction, and basic supportive care. Non-hematological toxicities were feasible.

**Figure 1.**
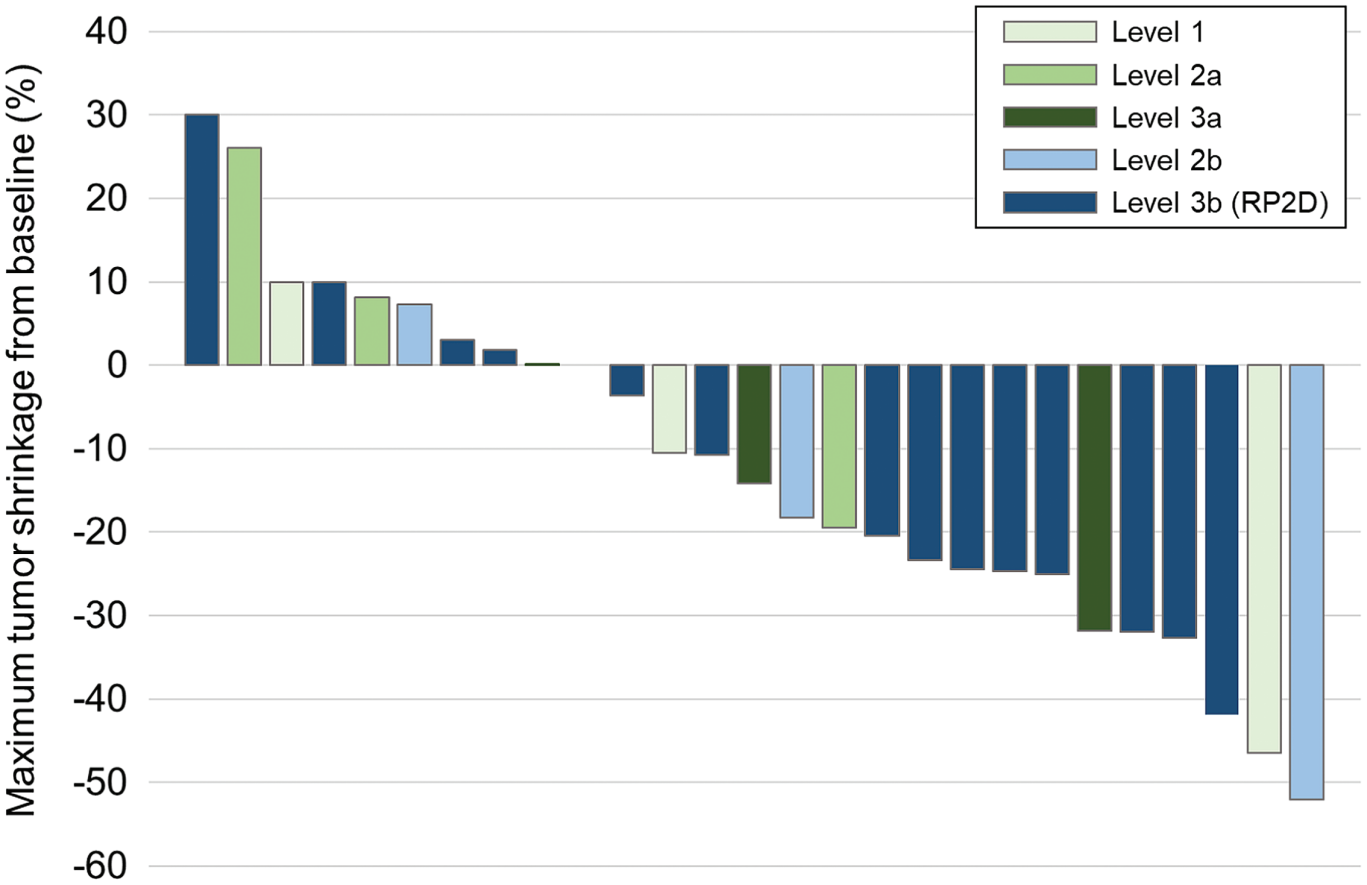
Waterfall plot: maximum tumor shrinkage from baseline.

**Figure 2.**
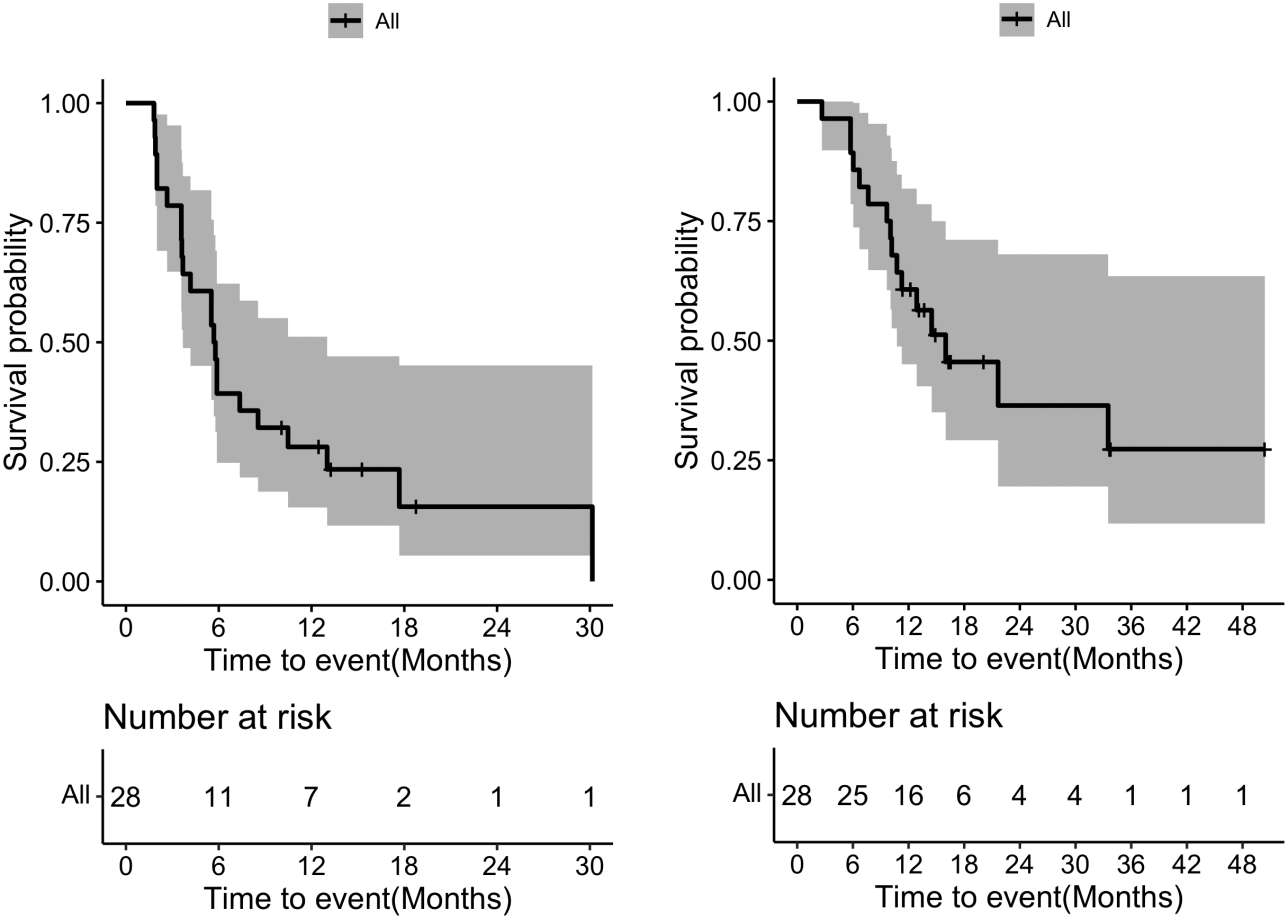
Kaplan-Meier plots: progression-free survival (left) and overall survival (right).

In the entire population, at a median follow-up time of 417 (range, 346-611) days, the overall response rate (ORR) was 21%; 6 patients achieved partial response in 28 patients. The disease control rate (DCR) was 79% (Fig. 1); 17 patients showed decrease in their target lesion sizes. The median PFS was 5.7 months (95% CI, 3.7-13.0 months) and the median OS was 16.0 (95% CI, 10.7-NA) months, respectively (Fig. 2).

Even with biweekly administration of FTD/TPI, the combination of FTD/TPI, IRI plus BEV produced a high rate of myelosuppression. The preliminary efficacy results showed comparable antitumor activity compared to the standard FOLFIRI regimen. Continued investigation of this regimen may be possible in other patient populations such as metastatic gastric cancer.

**Table:** 

Trial Information	
Disease	Colorectal cancer
Stage of disease/treatment	Metastatic/advanced
Prior therapy	More than one prior regimen
Type of study	Phase I; 3 + 3
Primary endpoint	Recommended phase II dose, frequency of febrile neutropenia
Secondary endpoints	Safety, efficacy
Investigator’s analysis	Active but results overtaken by other developments

## Additional Details of Endpoints or Study Design

### Study design:

This study was conducted using an open-label, nonrandomized, 2-part phase Ib design and was carried out at 2 centers. The study population consisted of a dose escalation cohort (part 1) to establish the recommended phase II dose (RP2D) for biweekly administration of the combination of FTD/TPI, IRI, and BEV, and an expansion cohort (part 2) for further investigation of safety and provision of a preliminary assessment of the antitumor activity of the combination regimen at the RP2D in patients with mCRC in whom fluoropyrimidine and oxaliplatin treatment had failed.

### Eligibility criteria:

Eligible patients were aged 20-75 years with histologically confirmed CRC, failure of or intolerance to fluoropyrimidine and oxaliplatin treatment (those who showed relapse fewer than 6 months after administration of the final dose of adjuvant chemotherapy were also eligible), and no prior therapy with FTD/TPI and IRI. Additional inclusion criteria included an Eastern Cooperative Oncology Group (ECOG) performance status of 0-1, adequate organ function (bone marrow, liver, and kidney), and at least one measurable metastatic lesion assessed according to the revised Response Evaluation Criteria In Solid Tumors (RECIST), version 1.1.

### Dose escalation:

The dose escalation cohort was formulated based on a traditional 3 + 3 design in which patients in sequential dose-level cohorts received increasing doses of FTD/TPI (25, 30, or 35 m/m2/dose, twice per day for days 1-5) and IRI (150 or 180 mg/m^2^ on day 1) together with a fixed dose of BEV (5 mg/kg on day 1) every 2 weeks.

At least 3 evaluable patients were treated with each dose; 6 patients were treated at the RP2D. An increase in subsequent doses was allowed only after the previous dose was determined to be safe as per protocol. Intra-patient dose escalation was not permitted. The RP2D was defined as the highest dose at which 1/3 or 2/6 of the treated evaluable patients experienced dose-limiting toxicity (DLT) during cycles 1 or 2. The expansion cohort was initiated as soon as the RP2D had been established; up to 15 patients were enrolled in parallel and administered FTD/TPI and IRI with BEV at the RP2D.

## Evaluation

The primary endpoint was determination of the RP2D in the dose escalation cohort and frequency of febrile neutropenia in the expansion cohort. Adverse events (AEs) were graded according to the National Cancer Institute (NCI) Common Terminology Criteria for Adverse Events version 4.03. A DLT was defined as a cycle of one or 2 AEs that met one of the following criteria: grade 4 neutropenia lasting >7 days; grade ≥3 febrile neutropenia, grade 4 thrombocytopenia, or grade 3 thrombocytopenia requiring platelet transfusion; drug-related toxicity resulting in a >14-day delay in starting cycle 2 or 3; and grade ≥3 non-hematologic toxicity except for the following AEs: (1) grade ≥3 gastrointestinal symptoms that could be controlled by supportive care; (2) electrolyte and γGTP abnormalities; and (3) grade ≥3 hypertension that could be controlled. The population assessed for treatment safety included all patients who received one or more doses of the study drug.

Antitumor responses were evaluated using RECIST version 1.1; each investigator performed tumor assessments at baseline and every 8 weeks and 12 weeks after week 32. Patients who had completed one or more treatment cycles and had undergone radiological/clinical progression assessments were evaluable for efficacy determination.

### Statistical analyses:

Descriptive statistics were used to summarize patient characteristics and safety and efficacy of the treatment. OS and PFS were estimated using the Kaplan-Meier method. The median OS and PFS and their 95% CIs were analyzed. Log-rank tests were used to compare differences in survival rates. All tests were 2-sided with a significance level of 0.05. All data summaries and listings were produced using R version 4.0.5 (Vienna, Austria).

**Table:** 

Drug Information	
**Generic/Working name**	Trifluridine/Tipiracil
**Company Name**	Lonsurf
**Drug Type**	Antimetabolites
Drug Class	Small molecule
Dose	25-35
**Unit**	mg/m^2^
**Route**	Oral (p.o.)
Schedule of Administration	Twice daily, days 1-5, every 2 weeks
Generic/Working name	Irinotecan
Drug Type	DNA topoisomerase I inhibitor
Drug Class	Small molecule
Dose	150-180
Unit	mg/m^2^
Route	i.v.
Schedule of Administration	day 1
Generic/Working name	Bevacizumab
Drug Type	VEGF inhibitor
Drug Class	Antibody
Dose	5
Unit	mg/kg
Route	i.v.
Schedule of Administration	day 1

**Table:** 

Drug Escalation Table			
Dose level	Trifluridine/Tipiracil (mg/m2)	Irinotecan (mg/m2)	Bevacizumab (mg/kg)
Level 1	25	180	5
Level 2a	30	180	5
Level 3a	35	180	5
Level 2b	30	150	5
**Level 3b**	35	150	5

**Table:** 

Patient Characteristics	
Number of patients, male	16
Number of patients, female	12
**Stage**	IV
Age: Median (range)	67.5 (33-74) years
Number of prior systemic therapies	1 (fluoropyrimidine plus oxaliplatin)
Performance Status: ECOG	0: 21 1: 7

**Table:** 

Dose Limiting Toxicity					
Dose Level	Dose of FTD/TPI (mg/m2)	Dose of irinotecan (mg/m2)	Number Enrolled	Number DLT	DLT information
**Level1**	**25**	**180**	**3**	**1**	**Febrile neutropenia**
**Level2a**	**30**	**180**	**3**	**2**	**Febrile neutropenia**
**Level3a**	**35**	**180**	**3**	**0**	**-**
**Level2b**	**30**	**150**	**3**	**1**	**GI perforation**
**Level3b**	**35**	**150**	**6**	**2**	**Treatment delay due to nausea** **ALT increased**

**Table:** 

Secondary Assessment Method	
Title	Response rate, Disease control rate, Progression-free survival, Overall survival
Number of patients screened	28
Number of patients enrolled	28
Number of patients evaluable for toxicity	28
Number of patients evaluated for efficacy	28
Evaluation Method	RECIST 1.1
Response assessment, CR	0 (0%)
Response assessment, PR	6 (21%)
Response assessment, SD	16 (57%)
Response assessment, PD	6 (21%)
Median duration assessment, PFS	5.7 months (95% CI: 3.7-13)
Median duration assessment, OS	16 months (10.7-NA)

## Adverse Events

**Table:** 

Adverse events, n (%)	All (n=28) Any Grade	All (n=28) Grade 3-4	RP2D (n=16) Any Grade	RP2D (n=16) Grade 3-4
Leukopenia	28 (100%)	13 (46%)	16 (100%)	9 (63%)
Neutropenia	28 (100%)	24 (86%)	16 (100%)	14 (86%)
Anemia	28 (100%)	3 (11%)	16 (100%)	1 (6%)
Febrile neutropenia	2 (7%)	2 (7%)	0	0
Infection	8 (29%)	2 (7%)	7 (44%)	0
Fatigue	23 (82%)	0	13 (81%)	0
Anorexia	20 (71%)	2 (7%)	10 (63%)	1 (6%)
Nausea	16 (57%)	0	7 (44%)	0
Vomiting	5 (18%)	0	1 (6%)	0
Diarrhea	12 (43%)	2 (7%)	7 (44%)	1 (6%)
Oral mucositis	6 (21%)	0	2 (13%)	0
Alopecia	17 (61%)	0	8 (50%)	0
Hypertension	15 (54%)	2 (7%)	8 (50%)	0
Proteinuria	18 (64%)	1 (4%)	11 (69%)	1 (6%)
Bleeding	8 (29%)	0	3 (19%)	0
Total bilirubin	4 (14%)	0	2 (13%)	0
Aspartate aminotransferase	19 (48%)	0	12 (75%)	0
Alanine aminotransferase	13 (46%)	3 (11%)	9 (56%)	2 (13%)
Creatinine	1 (4%)	0	0	0

## Assessment, Analysis, and Discussion

**Table:** 

Completion	Study completed
Investigator’s Assessment	Active but results overtaken by other developments

This phase Ib study indicated that combining IRI and BEV with biweekly FTD/TPI is safe for mCRC treatment. In Japanese mCRC patients pretreated with fluoropyrimidine and oxaliplatin, this regimen showed modest efficacy in this pilot study. To the best of our knowledge, this is the first study to evaluate such a triple combination with biweekly administration of FTD/TPI in Asian patients.

Five DLTs occurred in the dose escalation part of the study. We determined that the RP2D could be defined as FTD/TPI 35 mg/m^2^ (administered twice per day on days 1-5) plus IRI 150 mg/m^2^ (given on day 1 of a 14-day cycle) combined with BEV (5.0 mg/kg) for 2 weeks. A previous phase I study of combined FTD/TPI and IRI in Japanese patients reported a recommended dose of FTD/TPI 25 mg/m^2^ (administered twice daily on days 1-5 and 8–12 of a 28-day cycle) plus biweekly IRI 150 mg/m^2^.^3^ This dose was lower than that used in our study that used biweekly administration. Compared to a previous phase I study, our trial documented a similarly high rate of neutropenia, but a lower incidence of febrile neutropenia (33% in the previous study vs. 7% of the whole population in our current study). A recent report indicated that a biweekly schedule of FTD/TPI combined with BEV is expected to maintain dose intensity and antitumor activity but with less drug-related neutropenia (although data for a direct comparison are lacking).^4^ In addition, recent dose escalation studies of biweekly FTD/TPI combined with oxaliplatin determined a maximum tolerated dose of FTD/TPI 35 mg/m^2^, as in our study. In contrast, Varghese et al. reported on a phase 1 study of biweekly FTD/TPI, IRI, and BEV and showed that the MTD was FTD/TPI 25 mg/m^2^ plus IRI 180 mg/m^2^.^5^ This differs from our RP2D. Comparing the above study with the current study, grade 3-4 neutropenia was found to occur more frequently in our trial (42% vs. 86%) in the recommended dose cohort; however, a similar incidence of non-hematological toxicities was observed. This may be partially due to differences in ethnicity, IRI dose, and definition of DLT. Nonetheless, future studies of FTD/TPI combination regimens will be performed using biweekly FTD/TPI administration.

The triple drug combination was tolerable, and no drug-related AE was observed other than those that might be expected. Most drug-related AEs were grade 3-4 hematological events that could be managed by delay in the treatment schedule, dose reduction, and basic supportive care. Gastrointestinal toxicities such as anorexia and diarrhea were mainly grade 1 or 2 and related to FTD/TPI and IRI, which was also within our expectations. In addition, grade 3 hypertension, proteinurea, and perforation related to BEV were observed in <10% of our patients. AEs were managed using dose reduction and dose delay, with only 2 treatment-related AEs resulting in treatment discontinuation. Thus, the overall safety profile of this regimen is consistent with the expected toxicities of the individual agents, with no unanticipated safety issue.

The preliminary efficacy results showed modest antitumor activity in this trial. We reported an ORR of 19% and PFS of 7.1 months in the RP2D population, which is numerically better compared to previous trials of those for other conventional chemotherapies, including FOLFIRI in combination with anti-VEGF as second-line therapy.^6-9^ Considering the differences in the mechanisms of action of FTD/TPI and 5-FU and the antitumor activity of FTD/TPI even in a 5-FU resistant population, it is logical to apply FTD/TPI in combination with IRI than to continue administering 5-FU as second-line therapy after failure of fluoropyrimidine treatment. IRI and FTD/TPI are used sequentially according to the current standard of care. However, not all patients can receive FTD/TPI therapy owing to unexpected disease progression or worsening condition after second-line therapy with IRI. Based on our results, combination therapy with FTD/TPI and IRI is also reasonable, allowing for administration of both the key drugs in the same treatment line.

The limitation of this study is that it was a nonrandomized prospective trial with a small sample size. In particular, because the PPTD population consisted of only 16 patients, it was difficult to accurately assess efficacy in such a heterogeneous group with various prognostic factors such as RAS status and previous treatment with biologics. Given the study design, it was also difficult to determine the most appropriate IRI dose between 150 mg/m^2^ and 180 mg/m^2^. This was at least partly because all except one patient had a wild-type or heterozygous UGT1A1 genotype, which is assumed to confer a lower risk of neutropenia caused by IRI than homozygosity for UGT1A1*6 or UGT1A1*28.^10^

In conclusion, this dose escalation/expansion study indicated that treatment with a triple combination of FTD/TPI, IRI, and BEV has moderate antitumor activity with severe but manageable myelotoxicity for mCRC after failed standard first-line therapy with fluoropyrimidines and oxaliplatin. Continued investigation of this regimen may be possible in other patient populations such as metastatic gastric cancer.

## Data Availability

The data underlying this article will be shared on reasonable request to the corresponding author.
